# EXAMINING FRAILTY SUBDOMAINS (PHYSICAL, PSYCHOLOGICAL, COGNITIVE, SOCIAL) IN COMMUNITY-LIVING ADULTS 45–85

**DOI:** 10.1093/geroni/igad104.2793

**Published:** 2023-12-21

**Authors:** Lauren Griffith, Graciela Muniz-Terrera, Edwin van den Heuvel, Jayati Khattar, David Hogan, Megan O’Connell, Melanie Levasseur, Parminder Raina

**Affiliations:** McMaster University, Hamilton, Ontario, Canada; Ohio University, Athens, Ohio, United States; Eindhoven University of Technology, Eindhoven, Noord-Brabant, Netherlands; McMaster University, Hamilton, Ontario, Canada; Univeresity of Calgary, Calgary, Alberta, Canada; University of Saskatchewan, Saskatoon, Saskatchewan, Canada; Université de Sherbrooke, Sherbrooke, Quebec, Canada; McMaster University, Hamilton, Ontario, Canada

## Abstract

Frailty prevalence estimates vary considerably (4.0% to 59.1% in one review). Understanding these differences and what drives frailty among individuals could lead to more focused interventions. Breaking frailty into subdomains and exploring their relationships is one approach to this. A 127-item overall Frailty Index (FI) based on data collected on Canadian Longitudinal Study on Aging (CLSA) Comprehensive Cohort participants (n = 30,097 community-living adults aged 45-85 at baseline) was used to create physical, psychological, cognitive, and social subdomain-specific FIs. Each FI was divided into quintiles with the highest 20% (Q5) considered the frailest. We assessed subdomain FI quintile concordance (Goodman and Kruskal’s gamma), described the joint distribution of those most frail (Q5), and estimated the association between sex and age with Q5 subdomain FIs membership (logistic regression). The concordance among FI subdomain quartiles was low. The highest gammas (0.25-0.36) were between the physical/psychological and psychological/cognition subdomains. Concordance was generally higher in females who had higher odds of Q5 membership for overall (1.5), physical (1.7), psychological (1.6) and social frailty (1.1), and lower odds for cognitive frailty (0.7). The odds of Q5 membership increased with age for overall frailty and all subdomains except psychological frailty, which decreased with age. Our results suggest low concordance among frailty subdomains and the relationship between Q5 membership with sex and age differed by subdomain. These data may help to better target frailty interventions, but longitudinal data are needed to explore both the time course and interrelationships across subdomains.

